# An Isolated Unilateral Condylar Head Fracture in an Adult Female: A Case Report

**DOI:** 10.7759/cureus.62813

**Published:** 2024-06-21

**Authors:** Gauri Sharma, Deepankar Shukla, Nitin Bhola

**Affiliations:** 1 Oral and Maxillofacial Surgery, Sharad Pawar Dental College and Hospital, Datta Meghe Institute of Higher Education and Research, Wardha, IND

**Keywords:** closed reduction, mandible fracture, mandibular head fracture, condylar head fracture, condyle fracture, mandibular fracture

## Abstract

Mandible fractures are one of the most common facial fractures. Within the mandible, the condylar process fractures have the highest frequency of occurrence. This fracture type is associated with cases of assaults and falls. Fractures of the condylar head are frequently missed on clinical examination if the ramus height shortening is absent. These types of fractures have a higher incidence in the pediatric population. Condyles tend to fracture with other anatomical subsites of the mandible. The isolated fracture of a single condylar component is less common. This report highlights the unusual case of an isolated unilateral condylar head fracture in an adult female following a road traffic accident (RTA). This case report attempts to discuss the incidence rate of such types of fractures and the controversies surrounding them.

## Introduction

The mandible is the second most commonly fractured bone in the facial skeleton after the nasal bone [1,2]. The mandible has different anatomical sub-sites that have varied susceptibility to fractures. Most authors agree that the condylar region is the most frequently fractured site within the mandible [2-7]. There is a strong correlation between gender and incidence of condylar fractures. The fracture of this mandibular subsite is three times more prevalent in males when compared with females [8].

The mandibular bone typically fractures in a direct and indirect type of mechanism of injury that leads to multiple fractured sites [9,10]. When the fracture of the mandible occurs with a single fracture line, the mandibular angle region is the most commonly affected subsite [11]. The isolated fracture of a unilateral condyle is an unusual phenomenon. The literature states the incidence of such fractures at 1%-17% [9,11-15].

Intracapsular fractures are more commonly present in individuals below 18 years of age. This type of fracture is commonly missed on initial evaluation and plain X-rays [9,10]. Management of such a fracture is traditionally done using closed reduction with satisfactory results. The atypical presentation of an isolated unilateral intracapsular condylar fracture in an adult female represents an unusual fracture pattern that is rare in routine clinical practice. This report highlights one such case and discusses the various treatment modalities for condylar head fractures.

## Case presentation

A 30-year-old female patient reported to the Outpatient Department of Oral and Maxillofacial Surgery with complaints of right ear pain and reduced mouth opening for one month. The patient gave a history of road traffic accident (RTA) one month back. After the RTA, the patient had reported to a nearby government hospital. There the patient underwent suturing of a chin laceration using non-absorbable silk sutures. The patient was discharged with a five-day course of antibiotics, with analgesics, and no further treatment was done. The patient reported back after seven days, and suture removal of the laceration over the chin was done. The patient had lingering pain near the right ear, for which she was prescribed a further three-day course of analgesics.

After 15 days of the RTA, the patient’s right ear pain did not subside. She then reported to an Ear, Nose, and Throat (ENT) surgeon, who could not find any abnormality in the ear upon examination. The ENT specialist referred the patient to a dental practitioner for further evaluation. The patient visited a general dentist who undertook an orthopantomogram (OPG) and advised the patient to report to our facility for management.

**Figure 1:**
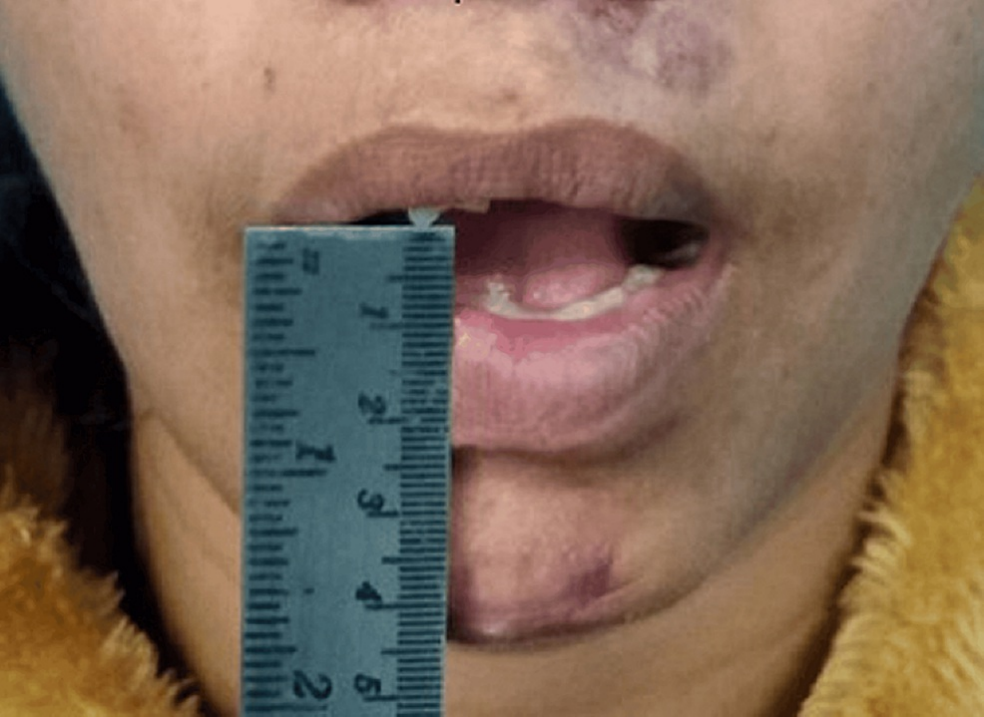
Reduced mouth opening of the patient

**Figure 2:**
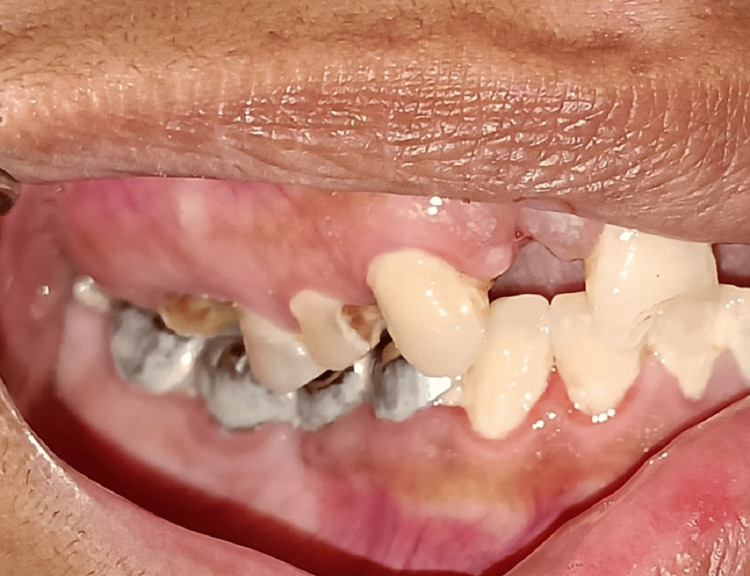
The occlusion of the patient on the right side

**Figure 3:**
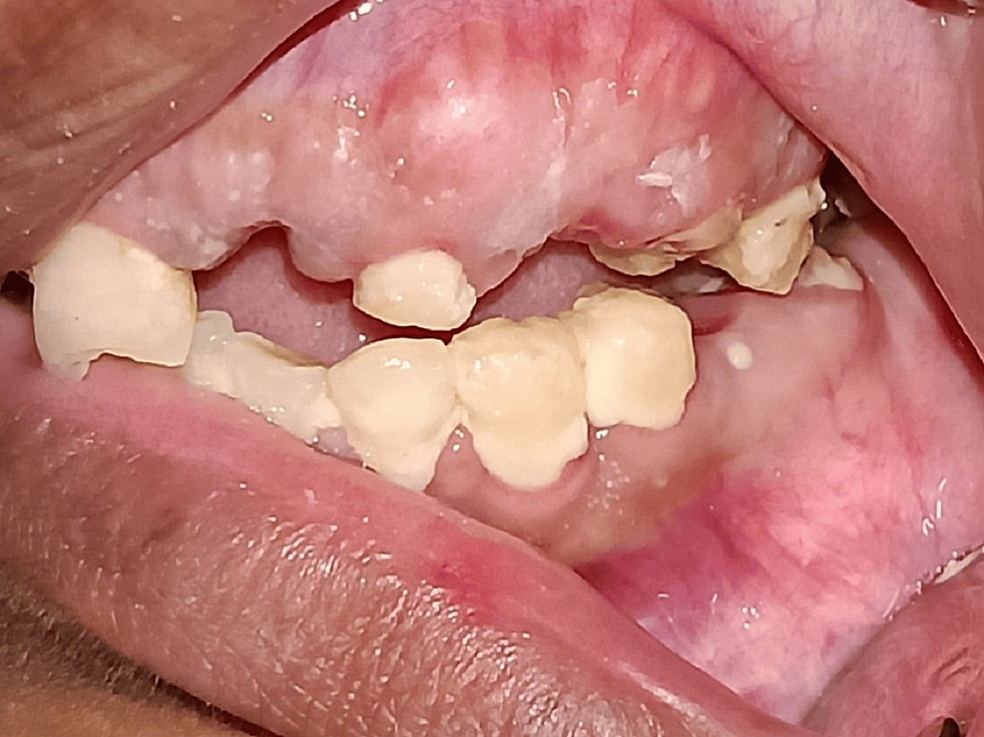
The occlusion of the patient on the left side

When we clinically examined the patient, there was clicking and tenderness over the right pre-auricular region. The patient’s maximum mouth opening was 11 mm, with bilaterally stable occlusion (Figures 1-3).

**Figure 4:**
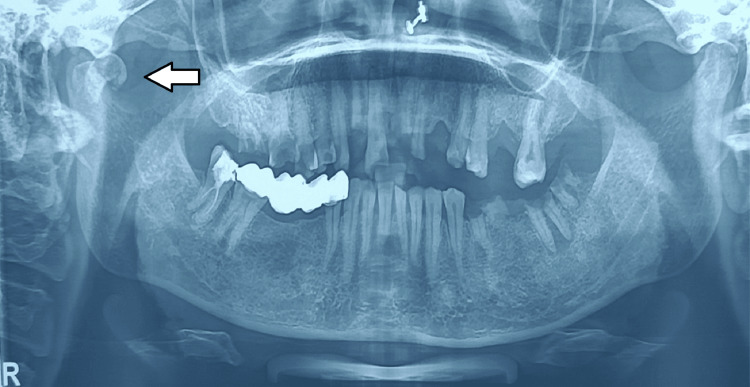
The OPG of the patient shows a right intracapsular sagittal fracture of the mandibular condyle OPG: orthopantomogram

The OPG of the patient showed features that were suggestive of a right intracapsular fracture in the sagittal plane (Figure 4). The contralateral condyle was intact.

No other facial bone or long bone fracture was present. It was noted that the fracture was one month old and present in the head of the condyle. After a thorough evaluation, the treating surgeons at our facility decided to manage the fracture conservatively. They advised the patient to maintain a soft diet, perform passive mouth-opening exercises, undergo regular physiotherapy, and report for weekly follow-up.

## Discussion

Condyles are the most common anatomical region of the mandible to undergo fracture during trauma [2-7]. The prevalence of condylar fractures strongly correlates with the male gender [8]. Condylar head fractures are more frequently reported in pediatric patients [15]. The biomechanics of the mandible typically result in direct and indirect types of injuries, leading to more than one fracture site [9,10]. Considering these facts, the isolated and unilateral fracture of the condylar head in an adult female patient is an unusual finding.

Mandibular head fractures are possibly underdiagnosed as they are overlooked on clinical examination and missed on plain X-rays [9,10]. There is no defined treatment protocol for the management of intracapsular condylar fractures in the adult population. The difficulty in attaining adequate access and surgical exposure is a predominant reason for the unpopularity of open reduction of condylar head fractures. The landmark study by Zide and Kent states that the open reduction followed by internal fixation of condylar fractures should be performed in cases of condylar displacement in the anterior cranial fossa or outside of the temporomandibular joint capsule [16]. Both of these conditions were not present in this case, which was the reason for our choice of conservative management of the fracture.

Two literature analyses on the ideal treatment plan for adult condylar fractures concluded that the management of these fractures is best done on a case-by-case basis according to the judgment of the treating surgeon [17,18]. Besides the criteria highlighted by Zide and Kent [16], several other factors such as occlusion and degree of mandibular movements must be considered [17,18]. The results of a meta-analysis for the best management practices for condylar fractures concluded the use of the open reduction technique in cases of low condylar fracture, with severe malocclusion and/or dislocation [19]. This further supports our decision to treat the condylar head fracture conservatively. The study further states that the outcomes of non-surgical management in terms of improvement in mouth opening were superior to those of surgical treatment [19]. Unfortunately, we cannot verify this finding as we lost the patient to follow-up.

The latest developing school of thought is directing focus toward exploring open approaches for intracapsular fractures [20,21]. The surgical treatment approach for condylar head fractures is still at a nascent stage and needs further studies before being adopted as the first line of treatment.

## Conclusions

This case report depicts an unusual condylar fracture pattern in an adult female patient. Condyle fractures are most common in men, secondary to assaults and falls. Fractures of the condylar head are difficult to diagnose based on clinical examination alone. Typically, mandibular fractures are associated with more than one fracture site due to the coup-contre coup mechanism of injury. The occurrence of an isolated and unilateral condylar head fracture is a rare phenomenon in an adult, as they are commonly associated with children.
